# Allosteric Modulation of the Calcium-sensing Receptor Rectifies Signaling Abnormalities Associated with G-protein α-11 Mutations Causing Hypercalcemic and Hypocalcemic Disorders*

**DOI:** 10.1074/jbc.M115.696401

**Published:** 2016-03-18

**Authors:** Valerie N. Babinsky, Fadil M. Hannan, Caroline M. Gorvin, Sarah A. Howles, M. Andrew Nesbit, Nigel Rust, Aylin C. Hanyaloglu, Jianxin Hu, Allen M. Spiegel, Rajesh V. Thakker

**Affiliations:** From the ‡ Radcliffe Department of Medicine, University of Oxford, Oxford OX3 7LJ, United Kingdom,; § Department of Musculoskeletal Biology, University of Liverpool, Liverpool L69 3GA, United Kingdom,; ¶ Biomedical Sciences Research Institute, Ulster University, Coleraine BT52 1SA, United Kingdom,; ‖ Sir William Dunn School of Pathology, University of Oxford, Oxford OX1 3RE, United Kingdom,; ** Department of Surgery and Cancer, Institute of Reproductive Biology and Development, Imperial College London, London W12 0NN, United Kingdom,; ‡‡ Laboratory of Bioorganic Chemistry, NIDDK, National Institutes of Health, Bethesda, Maryland 20892, and; §§ Albert Einstein College of Medicine, Bronx, New York 10461

**Keywords:** calcium, drug action, G protein, genetic disease, parathyroid hormone, GNA11, autosomal dominant hypocalcemia, familial hypocalciuric hypercalcemia, uveal melanoma

## Abstract

Germline loss- and gain-of-function mutations of G-protein α-11 (Gα_11_), which couples the calcium-sensing receptor (CaSR) to intracellular calcium (Ca^2+^*_i_*) signaling, lead to familial hypocalciuric hypercalcemia type 2 (FHH2) and autosomal dominant hypocalcemia type 2 (ADH2), respectively, whereas somatic Gα_11_ mutations mediate uveal melanoma development by constitutively up-regulating MAPK signaling. Cinacalcet and NPS-2143 are allosteric CaSR activators and inactivators, respectively, that ameliorate signaling disturbances associated with CaSR mutations, but their potential to modulate abnormalities of the downstream Gα_11_ protein is unknown. This study investigated whether cinacalcet and NPS-2143 may rectify Ca^2+^*_i_* alterations associated with FHH2- and ADH2-causing Gα_11_ mutations, and evaluated the influence of germline gain-of-function Gα_11_ mutations on MAPK signaling by measuring ERK phosphorylation, and assessed the effect of NPS-2143 on a uveal melanoma Gα_11_ mutant. WT and mutant Gα_11_ proteins causing FHH2, ADH2 or uveal melanoma were transfected in CaSR-expressing HEK293 cells, and Ca^2+^*_i_* and ERK phosphorylation responses measured by flow-cytometry and Alphascreen immunoassay following exposure to extracellular Ca^2+^ (Ca^2+^*_o_*) and allosteric modulators. Cinacalcet and NPS-2143 rectified the Ca^2+^*_i_* responses of FHH2- and ADH2-associated Gα_11_ loss- and gain-of-function mutations, respectively. ADH2-causing Gα_11_ mutations were demonstrated not to be constitutively activating and induced ERK phosphorylation following Ca^2+^*_o_* stimulation only. The increased ERK phosphorylation associated with ADH2 and uveal melanoma mutants was rectified by NPS-2143. These findings demonstrate that CaSR-targeted compounds can rectify signaling disturbances caused by germline and somatic Gα_11_ mutations, which respectively lead to calcium disorders and tumorigenesis; and that ADH2-causing Gα_11_ mutations induce non-constitutive alterations in MAPK signaling.

## Introduction

Guanine nucleotide-binding protein (G-protein)^4^ α-11 (Gα_11_) is a major intracellular signaling partner of the cell-surface G-protein-coupled calcium (Ca^2+^)-sensing receptor (CaSR), which plays a pivotal role in the parathyroid and renal regulation of extracellular Ca^2+^ (Ca^2+^*_o_*) concentrations (1, 2). Gα_11_ belongs to the G_q/11_ class of G-proteins that enhance phospholipase C activity (3), thereby leading to an accumulation of inositol 1,4,5-trisphosphate and rapid increase in intracellular Ca^2+^ (Ca^2+^_i_) concentrations (2, 4). These signal transduction events allow the CaSR to respond to small fluctuations in the prevailing Ca^2+^*_o_* concentration ([Ca^2+^]*_o_*) by inducing alterations in parathyroid hormone (PTH) secretion and urinary Ca^2+^ excretion (5).

The identification of germline heterozygous loss- and gain-of-function mutations of Gα_11_, which is encoded by the *GNA11* gene on chromosome 19p13.3, that lead to forms of familial hypocalciuric hypercalcemia (FHH) or autosomal dominant hypocalcemia (ADH), respectively, has demonstrated the importance of this G-protein subunit in Ca^2+^*_o_* homeostasis (1, 6, 7). FHH is a genetically heterogeneous disorder that is inherited as an autosomal dominant condition, which is characterized by lifelong elevations of serum Ca^2+^ concentrations in association with normal or mildly raised serum PTH levels and low urinary Ca^2+^ excretion (8). FHH is considered to represent a benign disorder, however some patients may develop symptomatic hypercalcemia, pancreatitis, or chondrocalcinosis (8). FHH type 1 (FHH1, OMIM #145980) is caused by loss-of-function mutations of the *CASR* gene (9), and FHH type 2 (FHH2, OMIM #145981) is caused by loss-of-function Gα_11_ mutations, which comprise a L135Q missense substitution and in-frame isoleucine deletion at codon 199 or 200 (I199/200del) that impair CaSR signal transduction and were identified in two unrelated probands and families (1). ADH is also genetically heterogeneous and caused by germline gain-of-function mutations of the *CASR* and *GNA11* genes, which lead to ADH types 1 (ADH1, OMIM #601198) and 2 (ADH2, OMIM #615361), respectively (1, 6–10). Approximately 50% of ADH patients develop hypocalcemic symptoms such as paraesthesia, carpo-pedal spasms, and seizures, and >35% of patients harbor ectopic calcifications within the kidneys or basal ganglia (1, 6, 7, 10). In contrast to germline gain-of-function Gα_11_ mutations, which affect Ca^2+^*_o_* homeostasis, somatic gain-of-function Gα_11_ mutations have been reported to lead to uveal melanoma, which is a primary intraocular tumor, by inducing constitutive up-regulation of proliferative signaling involving ERK, which is a component of the MAPK signaling pathway (11).

Compounds that selectively bind to the CaSR and allosterically modulate the function of this family C G-protein-coupled receptor (GPCR) represent a potential targeted therapy for patients with symptomatic forms of FHH and ADH. Indeed, cinacalcet, which is a licensed CaSR positive allosteric modulator, has been used effectively in FHH1 patients to manage symptomatic hypercalcemia and recurrent pancreatitis (12, 13). Furthermore, negative allosteric CaSR modulators, known as calcilytics, have been demonstrated to ameliorate hypocalcemia in mouse models of ADH1 (14, 15). The objective of this study was to undertake *in vitro* studies to determine whether allosteric modulators targeted to the CaSR may also rectify the loss- and gain-of-function associated with FHH2- and ADH2-causing germline Gα_11_ mutations, respectively, and the up-regulation of ERK phosphorylation caused by a uveal melanoma-associated somatic Gα_11_ mutation. In addition, this study evaluated whether germline ADH2-causing gain-of-function Gα_11_ mutations may constitutively activate MAPK signaling and thus pose a risk for the development of uveal melanomas.

## Experimental Procedures

### 

#### 

##### Cell Culture and Transfection

Functional studies of mutant Gα_11_ proteins were performed in HEK293 cells that stably expressed the CaSR (HEK-CaSR) (1, 16, 17). HEK293 cells endogenously express Gα_11_, and co-expression of mutant Gα_11_ proteins approximately represented the heterozygous state in FHH2 and ADH2 patients (1). The HEK-CaSR cell line was cultured in high-glucose DMEM (Invitrogen) supplemented with 10% fetal bovine serum (FBS) and 1% geneticin, as described (1, 16, 17). A high level of CaSR expression in these cells was confirmed by Western blot analysis using a mouse monoclonal antibody to human CaSR (ADD; Abcam, ab19347, 1:1,000) (1, 16). WT and mutant *GNA11*-pBI-CMV2 constructs were transiently transfected into HEK-CaSR cells using Lipofectamine 2000 (1, 16, 17). The bidirectional pBI-CMV2 cloning vector was used as it facilitated the co-expression of Gα_11_ and GFP (1, 16, 18). Expression of WT and mutant Gα_11_ proteins were determined by Western blot analysis using a mouse monoclonal anti-Gα_11_ antibody (SantaCruz Biotechnology, sc-390382, 1:750), and the membrane was re-probed with a polyclonal rabbit anti-α-tubulin antibody (Abcam, ab15246, 1:1000) as a loading control. Successful transfection was also confirmed by visualizing GFP fluorescence using an Eclipse E400 fluorescence microscope with a Y-FL Epifluorescence attachment and a triband 4,6-diamidino-2-phenylindole-FITC-Rhodamine filter, and images captured using a DXM1200C digital camera and NIS-Elements software (Nikon) (1, 16, 17).

Studies involving siRNA knockdown of endogenous Gα_11_ were undertaken in HEK293 cells that stably expressed WT or mutant Gα_11_ proteins (HEK-Gα_11_). The HEK-Gα_11_ cells were generated using HEK293 T-Rex-Flp-in stable cell lines (Life Technologies), as reported (19). WT and mutant *GNA11* constructs were cloned into the pcDNA5/FLP recombination target (FRT) expression vector (Life Technologies), and silent mutations introduced to render the constructs resistant to *GNA11*-targeted siRNA, thereby allowing investigation of the mutant Gα_11_ protein in the absence of endogenous WT Gα_11_. *GNA11* constructs were transiently transfected into T-Rex-Flp-in cells, and those cells expressing the Gα_11_ protein selected by culturing cells in medium containing Hygromycin (Gibco). The presence of the Gα_11_ protein and its resistance to siRNA was confirmed by Western blot analysis. Forty-eight hours prior to measuring Ca^2+^*_i_* responses, HEK-Gα_11_ cells were transiently transfected with the reported pEGFP-CaSR construct (9) and three different commercially available *GNA11*-targeted siRNA constructs (Trilencer-27 siRNA kit, catalogue number SR301839, Origene) or a commercially available scrambled siRNA (Trilencer-27 universal scrambled negative control siRNA duplex, catalogue number SR30004, Origene), and successful transfection confirmed by fluorescence microscopy, as described for pBI-CMV2-expressing HEK-CaSR cells (1, 16, 17).

##### Measurement of Ca^2+^_i_ Responses

The effect of allosteric CaSR modulators on cells expressing WT or mutant Gα_11_ proteins was assessed by a flow cytometry-based Ca^2+^*_i_* assay, as reported (1, 16, 17). In brief, 48 h after transfection, the cells were harvested, washed in Ca^2+^- and magnesium (Mg^2+^)-free Hank's balanced salt solution (HBSS) (Invitrogen), and loaded with 1 μg/ml indo-1-acetoxymethylester (Indo-1-AM) (Molecular Probes) for 1 h at 37 °C (1, 16, 17). Transfected cells were incubated with either a 20% aqueous solution of 2-hydroxypropyl-β-cyclodextrin (Sigma) (vehicle), or positive or negative CaSR allosteric modulators, known as cinacalcet or NPS-2143, respectively, at concentrations ranging from 10–40 nm for 1 h (15). Flow cytometry was performed with a Beckman Coulter MoFlo XDP equipped with JDSUY Xcyte UV Laser and a Coherent Sapphire 488 Laser using a 550LP dichroic mirror and 580/30 bandpass filter (17). Single cells were isolated and stimulated by sequentially adding Ca^2+^ to the Ca^2+^- and Mg^2+^-free HBSS to increase the [Ca^2+^]*_o_* in a stepwise manner from 0–15 mm. The range of [Ca^2+^]*_o_* used to activate CaSR signaling in HEK293 cells was not representative of physiological levels of serum ionized calcium, which are homeostatically maintained between 1.1–1.3 mm (20), but use of these Ca^2+^*_o_* concentrations *in vitro* allowed a comprehensive assessment of CaSR signaling responses, which included threshold responses (1–1.5 mm [Ca^2+^]*_o_*), half-maximal (EC_50_) responses (2–4 mm [Ca^2+^]*_o_*), and near-maximal responses (>10 mm), as reported (21). The baseline fluorescence ratio was measured for 2 min, the fluorescence ratio compared with the time was recorded, and data were collected for 2 min at each [Ca^2+^]*_o_*, as described (1, 16, 17). Cytomation Summit software was used to determine the peak mean fluorescence ratio of the transient response after each individual stimulus, which was expressed as a percentage normalized response (1, 16, 17). Concentration-response curves were generated using a 4-parameter non-linear regression curve-fit model (GraphPad Prism) to calculate the half-maximal (EC_50_) and area under the curve (AUC) mean ± S.E. responses for each separate experiment (17).

##### Measurement of ERK Phosphorylation

HEK-CaSR cells, transfected with WT or mutant Gα_11_ proteins for 24 h, were seeded in 48-well plates and cultured overnight in high glucose DMEM containing 10% FBS, prior to being incubated for 4 h with serum-free DMEM containing 0.5 mm Ca^2+^, 25 mm HEPES buffer with or without cinacalcet or NPS-2143 at 10–500 nm concentrations. Cells were stimulated for 4 min with pre-warmed serum-free DMEM that contained Ca^2+^ concentrations ranging from 0.5–10 mm, as reported (22), and lysed in Surefire lysis buffer. Alphascreen Surefire ERK phosphorylation assays were performed on whole cell lysates, as reported (23), and the fluorescence signal measured using a PHERAStar *FS* microplate reader (BMG Labtech) (23). ERK phosphorylation responses measured at each [Ca^2+^]*_o_* were normalized to the mean responses of WT expressing cells and expressed as a fold-change of responses obtained at basal (0.5 mm) [Ca^2+^]*_o_*.

##### Statistical Analysis

The Ca^2+^*_i_* and ERK phosphorylation responses of cells expressing WT or mutant Gα_11_ proteins were compared from a minimum of four experiments using the F-test and Mann-Whitney *U* test, respectively (1). All analyses were undertaken using GraphPad Prism (GraphPad), and are presented as mean ± S.E. A value of *p* < 0.05 was considered significant for all analyses.

## Results

### 

#### 

##### Effect of Cinacalcet on the Ca^2+^_i_ Responses of FHH2-associated Gα_11_ Mutations

**FIGURE 1.**
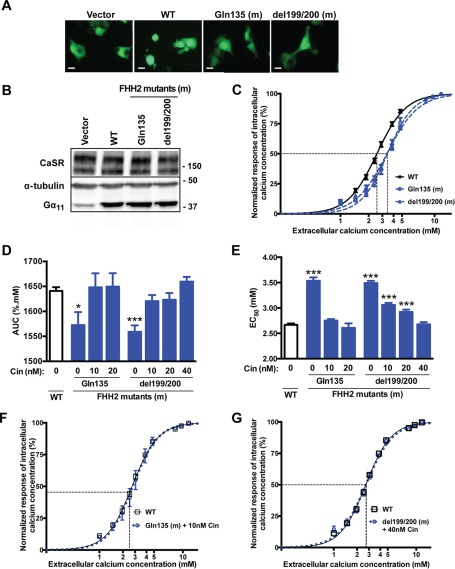
**Effect of cinacalcet on the Ca^2+^*_i_* responses of FHH2-associated Gα_11_ mutations.** *A*, fluorescence microscopy of HEK293 cells stably expressing CaSR (HEK-CaSR) and transiently transfected with WT or FHH2-associated (Gln-135 and del199/200) mutant (m) *GNA11*-pBI-CMV2-GFP constructs, or with vector only. GFP expression in these cells indicates successful transfection and expression by these constructs. Bar indicates 20 μm. *B*, Western blot analysis of whole cell lysates using antibodies to CaSR, α-tubulin, and Gα_11_. Transient transfection of WT or FHH2-associated mutant constructs resulted in over-expression of Gα_11_ when normalized to α-tubulin expression. *C* and *D*, Ca^2+^*_i_* response to changes in [Ca^2+^]*_o_* of HEK-CaSR cells transfected with WT or FHH2-associated Gα_11_ mutants. The Ca^2+^*_i_* responses to changes in [Ca^2+^]*_o_* are expressed as a percentage of the maximum normalized responses and shown as the mean ± S.E. of 6–16 assays from 2–4 independent transfections. The FHH2-associated Gα_11_ mutants (Gln-135 and del199/200) led to a rightwards shift of the concentration-response curves (*blue*) with significantly reduced AUC values (*blue bars*) when compared with WT Gα_11_ (in *C, black;* and in *D, open bar*), which harbors Leu and Ile residues at codons 135 and 199/200, respectively. *E*, FHH2-associated Gln-135 and del199/200 mutants (*blue bars*) are associated with significantly increased EC_50_ values compared with cells expressing WT Gα_11_ (*open bar*). The addition of 10 and 20 nm cinacalcet (*Cin*) decreased the EC_50_ values of cells expressing Gln-135 to values that were not significantly different from WT, whereas 40 nm cinacalcet was required to rectify the increased EC_50_ value of cells expressing the del199/200 mutant. *F* and *G*, addition of cinacalcet at 10 and 40 nm concentrations rectified the rightward shift in the concentration-response curves of the Gln-135 and del199/200 mutant Gα_11_ proteins, respectively. *, *p* < 0.05; ***, *p* < 0.0001.

The FHH2-associated L135Q and I199/200del Gα_11_ mutations have been reported to impair the sensitivity of CaSR-expressing cells to Ca^2+^*_o_* (1), and we hypothesized that cinacalcet-mediated allosteric activation of the CaSR would ameliorate the loss-of-function associated with germline mutations of Gα_11_, thereby rectifying the signal transduction abnormalities in cells expressing these FHH2-associated mutant Gα_11_ proteins. To investigate this hypothesis, WT or mutant *GNA11*-pBI-CMV2 constructs were transiently transfected in HEK-CaSR cells and the effect of cinacalcet on the responses of Ca^2+^*_i_* concentrations ([Ca^2+^]*_i_*) to alterations in [Ca^2+^]*_o_* was assessed. Expression of the CaSR and Gα_11_ was confirmed by fluorescence microscopy and/or Western blot analysis of whole-cell lysates (Fig. 1, *A* and *B*). CaSR expression, which was normalized by comparison to α-tubulin expression, did not differ between cells transfected with WT or FHH2-associated mutant *GNA11*-pBI-CMV2 vectors when compared with cells transfected with empty vector, whereas the expression of Gα_11_ was greater in cells transfected with WT or mutant constructs (Fig. 1*B*). HEK-CaSR cells transiently transfected with WT or mutant Gα_11_ proteins were exposed to varying [Ca^2+^]*_o_*, and measurement of [Ca^2+^]*_i_* responses by flow cytometry revealed the FHH2-associated Gln-135 and del199/200 Gα_11_ mutants to result in a rightward shift of the concentration-response curves (Fig. 1*C*) with a significant reduction in AUC values and increases in EC_50_ values (Gln-135 = 3.54 ± 0.07 mm, del199/200 = 3.49 ± 0.04 mm) compared with WT Gα_11_ (2.67 ± 0.03 mm; *p* < 0.0001) (Fig. 1, *D* and *E*), as reported (1). A dose-titration of cinacalcet in cells expressing the Gln-135 Gα_11_ mutant revealed this calcimimetic to act in a dose-dependent manner, with 10 and 20 nm drug concentrations significantly (*p* < 0.0001) reducing the Gln-135 mutant EC_50_ values to 2.75 ± 0.03 and 2.61 ± 0.09 mm, respectively (Fig. 1*E*). Indeed, 10 nm of cinacalcet induced a leftward shift of the mutant concentration-response curve, so that this was indistinguishable from that of WT-expressing cells (Fig. 1*F*). The addition of 10 and 20 nm cinacalcet lowered the EC_50_ values of cells expressing the del199/200 Gα_11_ mutant (Fig. 1*E*). However, despite the del199/200 mutant having an almost identical EC_50_ value to the Gln-135 Gα_11_ mutant protein, these cinacalcet doses were insufficient to rectify the loss-of-function associated with the del199/200 Gα_11_ mutant (Fig. 1*E*). Subsequently, when cinacalcet was added at a 40 nm concentration to cells expressing the del199/200 Gα_11_ mutant, this lowered the EC_50_ value to 2.68 ± 0.04 mm (Fig. 1*E*), so that the del199/200 mutant concentration-response curve overlapped with that of the WT Gα_11_ protein (Fig. 1*G*).

##### Effect of NPS-2143 on the Ca^2+^_i_ Responses of ADH2-associated Gα_11_ Mutations

**FIGURE 2.**
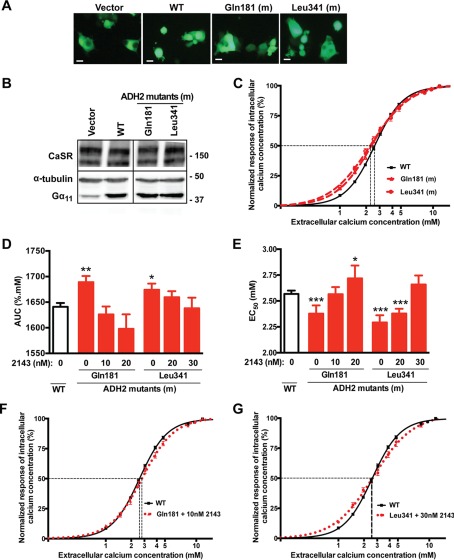
**Effect of NPS-2143 on the Ca^2+^*_i_* responses of ADH2-associated Gα_11_ mutations.** *A*, fluorescence microscopy of HEK293 cells stably expressing CaSR (HEK-CaSR) and transiently transfected with vector, WT, or ADH2-associated (Gln-181 and Leu-341) mutant (*m*) constructs. GFP expression in these cells indicates successful transfection and expression by these constructs. Bar indicates 20 μm. *B*, Western blot analysis of whole cell lysates using antibodies to CaSR, α-tubulin, and Gα_11_. Transient transfection of WT or ADH2-associated mutant constructs resulted in overexpression of Gα_11_ when normalized to α-tubulin expression. *C* and *D*, Ca^2+^*_i_* response to changes in [Ca^2+^]*_o_* of HEK-CaSR cells transfected with WT or ADH2-associated Gα_11_ mutants. The Ca^2+^*_i_* responses to changes in [Ca^2+^]*_o_* are expressed as a percentage of the maximum normalized responses and shown as the mean ± S.E. of 6–16 assays from 2–4 independent transfections. The ADH2-associated Gα_11_ mutants (Gln-181 and Leu-341) led to a leftwards shift of the concentration-response curves (*red*) with significantly increased AUC values (*red bars*) when compared with WT Gα_11_ (in *C, black;* and in *D, open bar*), which harbors Arg and Phe residues at codons 181 and 341, respectively. *E*, ADH2-associated Gln-181 and Leu-341 mutants (red bars) are associated with significantly reduced EC_50_ values compared with cells expressing WT Gα_11_ (*open bar*). The addition of 10 nm NPS-2143 (2143) increased the EC_50_ value of cells expressing the Gln-181 mutant so that this was not significantly different from WT, whereas 30 nm of NPS-2143 was required to rectify the reduced EC_50_ value of cells expressing the Leu-341 mutant. *F* and *G*, addition of NPS-2143 at 10 and 30 nm concentrations rectified the leftward shift in the concentration-response curves of the Gln-181 and Leu-341 mutant Gα_11_ proteins, respectively. *, *p* < 0.05; **, *p* < 0.01; ***, *p* < 0.0001.

We previously reported the germline R181Q and F341L Gα_11_ mutations to enhance the sensitivity of CaSR-expressing cells to Ca^2+^*_o_* (1), thereby giving rise to the hypocalcemic disorder of ADH2. To determine whether allosteric inhibition of the CaSR can rectify the gain-of-function associated with ADH2-causing Gα_11_ mutations, WT or ADH2-associated mutant *GNA11*-pBI-CMV2 vectors were transiently transfected into HEK-CaSR cells, and the responses of [Ca^2+^]*_i_* to alterations in [Ca^2+^]*_o_* assayed. Expression of the CaSR and Gα_11_ was demonstrated by fluorescence microscopy and/or Western blot analysis (Fig. 2, *A* and *B*). Western blot analysis confirmed an increase in the expression of Gα_11_ in cells transfected with WT or ADH2-associated mutant proteins, when compared with cells transfected with empty vector alone (Fig. 2*B*). An assessment of the Ca^2+^*_i_* responses of HEK-CaSR cells transiently transfected with WT or ADH2-associated mutant Gα_11_ proteins following stimulation with [Ca^2+^]*_o_*, demonstrated cells expressing the Gln-181 or Leu-341 mutants to induce a leftward shift of the concentration-response curves (Fig. 2*C*) with a significant increase in AUC values and reduction in EC_50_ values of 2.38 ± 0.08 and 2.29 ± 0.07 mm, respectively, compared with 2.57 ± 0.03 mm for WT-expressing cells (*p* < 0.0001) (Fig. 2, *D* and *E*), as previously reported (1). The addition of NPS-2143 to cells expressing the Gln-181 Gα_11_ mutant revealed a 10 nm concentration of this calcilytic compound to normalize the mutant EC_50_ value to 2.57 ± 0.07 mm (Fig. 2*E*), so that the concentration-response curve resembled that of WT Gα_11_ (Fig. 2*F*), whereas 20 nm NPS-2143 significantly increased the mutant EC_50_ value to 2.72 ± 0.12 mm when compared with WT expressing cells (Fig. 2*E*). In contrast to these studies involving the Gln-181 Gα_11_ mutant protein, the addition of 20 nm NPS 2143 to cells expressing the Leu-341 Gα_11_ mutant did not significantly alter the EC_50_. (Fig. 2*E*). Indeed, NPS-2143 at a concentration of 30 nm was required to increase the Leu-341 mutant EC_50_ value to 2.66 ± 0.09 mm and rectify the shift in the mutant concentration-response curve (Fig. 2, *E* and *G*).

##### Effect of CaSR Allosteric Modulators on the Ca^2+^_i_ Responses in Absence of Endogenously Expressed WT Gα_11_ Protein

**FIGURE 3.**
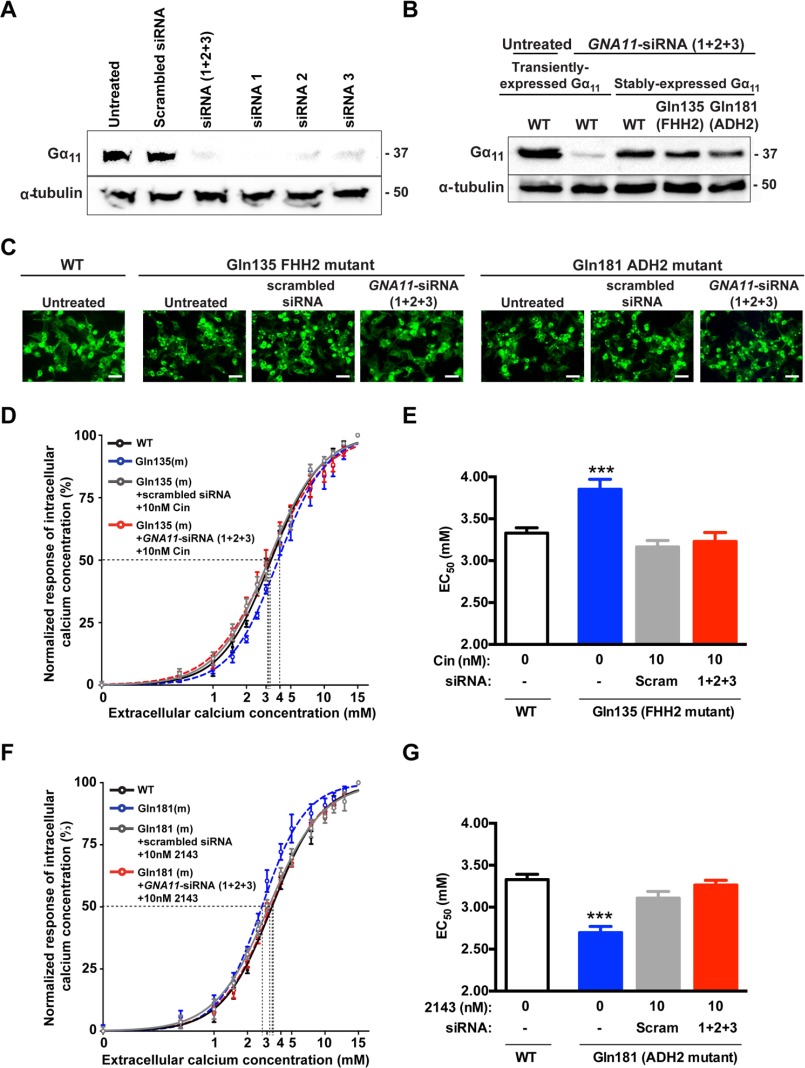
**Effect of cinacalcet and NPS-2143 on the Ca^2+^*_i_* responses of FHH2- and ADH2-associated Gα_11_ mutations following siRNA knockdown of endogenously expressed WT Gα_11_.** *A*, Western blot analysis of untransfected HEK293 cells, which express endogenous WT Gα_11_ only, and have been treated with either scrambled siRNA, or three different *GNA11-*targeted siRNAs (siRNAs 1–3), either alone or all together (siRNAs 1 + 2+3), and compared with untreated HEK293 cells. All three *GNA11*-targeted siRNAs, but not scrambled siRNA, reduced endogenous WT Gα_11_ expression. *B*, Western blot analysis showing combined effects of the three *GNA11*-targeted siRNAs on Gα_11_ protein expression in HEK293 cells transiently expressing WT Gα_11_ proteins and in HEK-Gα_11_ cells, which stably express WT or mutant Gα_11_ proteins. Use of *GNA11*-targeted siRNAs reduced Gα_11_ expression in HEK293 cells, but not in HEK-Gα_11_ cells, which are resistant to *GNA11*-targeted siRNA. *C*, fluorescence microscopy confirming transfection of untreated and siRNA-treated (scrambled or combined siRNAs 1 + 2+3) HEK-Gα_11_ cells with the pEGFP-CaSR construct. Bar indicates 50 μm. *D* and *E*, Ca^2+^*_i_* response to changes in [Ca^2+^]*_o_* of FHH2-associated mutant Gln-135 HEK-Gα_11_ cells following siRNA knockdown of endogenous WT Gα_11_. The Ca^2+^*_i_* responses to changes in [Ca^2+^]*_o_* are expressed as a percentage of the maximum normalized responses and shown as the mean ± S.E. of 4–5 independent transfections (*i.e.* biological replicates). The FHH2-associated Gα_11_ mutant (Gln-135) led to a rightward shift of the concentration-response curve (*blue*), with a significant increase in EC_50_ value compared with WT Gα_11_ (in *D, black;* and in *E, open bar*). The addition of 10 nm cinacalcet (*Cin*) normalized the EC_50_ values of cells in the presence of scrambled (gray) or *GNA11*-targeted siRNAs (siRNAs 1 + 2+3) (*red*). *F* and *G*, Ca^2+^*_i_* response to changes in [Ca^2+^]*_o_* of ADH2-associated mutant Gln-181 HEK-Gα_11_ cells following siRNA knockdown of endogenous WT Gα_11_. The ADH2-associated Gα_11_ mutant (Gln-181) led to a leftward shift of the concentration-response curve (*blue*), with a significant decrease in EC_50_ value compared with WT Gα_11_ (in *F, black;* and in *G, open bar*). The addition of 10 nm NPS-2143 (2143) normalized the EC_50_ value of cells in the presence of scrambled (*gray*) or *GNA11*-targeted siRNAs (siRNAs 1 + 2+3) (*red*). ***, *p* < 0.0001; −, nil; *scram*, scrambled.

To determine whether CaSR-targeted drugs rectify the Ca^2+^*_i_* responses of FHH2- and ADH2-mutant expressing cells by directly influencing mutant Gα_11_-signaling or by indirect effects on WT Gα_11_ protein that is endogenously expressed in HEK293 cells, siRNA knockdown of endogenous WT Gα_11_ was undertaken in HEK-Gα_11_ cells stably expressing WT, FHH2-associated Gln-135, or ADH2-associated Gln-181 mutant Gα_11_ proteins. Western blot analysis demonstrated that siRNA with a scrambled sequence did not alter endogenous WT Gα_11_ expression in untransfected HEK293 cells (Fig. 3*A*). In contrast, *GNA11*-targeted siRNA reduced endogenous WT Gα_11_ expression in untransfected HEK293 cells (Fig. 3*A*), and decreased the level of transiently expressed WT Gα_11_ in HEK293 cells (Fig. 3*B*), but did not affect the levels of stably expressed WT or mutant Gα_11_ proteins in HEK-Gα_11_ cells (Fig. 3*B*), which contained constructs with silent mutations that had rendered them resistant to *GNA11*-targeted siRNA. CaSR constructs were transiently transfected into HEK-Gα_11_ cells, and CaSR expression confirmed by fluorescence microscopy (Fig. 3*C*). The effects of cinacalcet or NPS-2143 on the Ca^2+^*_i_* responses of the FHH2- and ADH2-associated Gα_11_ mutants were assessed following knockdown of endogenous WT Gα_11_ using *GNA11*-targeted siRNAs (Fig. 3, *D–G*). These studies revealed that: 10 nm of cinacalcet could rectify the rightward shift in the concentration-response curve and lower the significantly raised EC_50_ of the FHH2-associated Gln-135 Gα_11_ mutant from a value of 3.85 ± 0.12 mm to values of 3.23 ± 0.1 mm and 3.17 ± 0.08 mm, respectively, in the presence of *GNA11*-targeted or scrambled siRNA (Fig. 3, *D* and *E*), so that these values were not significantly different from HEK-Gα_11_ cells stably expressing WT Gα_11_ (EC_50_ = 3.33 ± 0.06 mm); and that 10 nm of NPS-2143 could normalize the leftward shift of the concentration-response curve and increased the EC_50_ of the ADH2-associated Gln-181 Gα_11_ mutant from a value of 2.70 ± 0.07 mm to values of 3.26 ± 0.06 mm and 3.11 ± 0.08 mm, respectively, in the presence of *GNA11*-targeted or scrambled siRNA (Fig. 3, *F* and *G*), so that these values were not significantly different from WT-expressing HEK-Gα_11_ cells. Thus, these results show that CaSR-targeted drugs can influence the signaling responses of downstream mutant Gα_11_ proteins.

##### Effect of ADH2-associated Gα_11_ Mutants on MAPK Signaling

**FIGURE 4.**
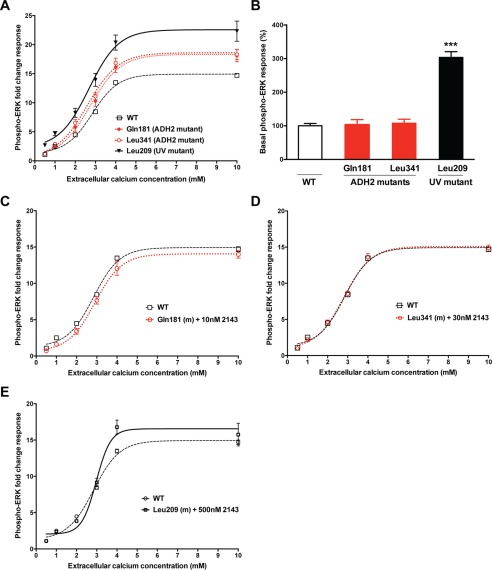
**Phospho-ERK responses of Gα_11_ mutations associated with ADH2 or uveal melanoma.** *A*, phospho-ERK response to changes in [Ca^2+^]*_o_* was measured by quantitative immunoassay (Alphascreen) in HEK-CaSR cells transiently transfected with WT or ADH2-associated Gα_11_ mutants (Gln-181 and Leu-341), or the uveal melanoma (UV)-associated Leu-209 Gα_11_ mutant protein. Phospho-ERK responses at each [Ca^2+^]*_o_* are expressed as a fold-change of the response of cells stimulated with basal (0.5 mm) [Ca^2+^]*_o_*, and are shown as the mean ± S.E. of 9–24 assays from 3–8 independent transfections. The uveal melanoma-associated Leu-209 Gα_11_ mutant is associated with significantly increased maximal phospho-ERK fold-change responses compared with WT (Gln-209) and the ADH2 mutant Gα_11_ proteins. The Gln-181 and Leu-341 mutants also induced significant increases in maximal phospho-ERK fold-change responses compared with WT Gα_11_, which harbors Arg and Phe residues at codons 181 and 341, respectively. *B*, quantification of the basal phospho-ERK responses shown in *A*. Values are expressed as a percentage of the WT basal phospho-ERK response. The uveal melanoma-associated Leu-209 Gα_11_ mutant induces a significant phospho-ERK elevation when exposed to basal 0.5 mm [Ca^2+^]*_o_*, whereas the basal phospho-ERK responses of the ADH2-associated Gα_11_ mutants are not significantly different compared with WT Gα_11_. *C–E*, addition of NPS-2143 (2143) at 10, 30, and 500 nm concentrations significantly decreased the phospho-ERK responses of the ADH2-associated Gln-181 and Leu-341 Gα_11_ mutants, and the UV-associated Leu-209 mutant Gα_11_ protein, respectively, to values that were not significantly different from WT Gα_11_. ***, *p* < 0.0001.

To investigate whether the germline R181Q and F341L ADH2-associated mutant Gα_11_ proteins may lead to constitutive up-regulation of MAPK signaling, WT and mutant *GNA11*-pBI-CMV2 vectors were transiently transfected into HEK-CaSR cells and fold-change ERK phosphorylation (phospho-ERK) responses assessed following exposure to varying [Ca^2+^]*_o_*. The effects of the ADH2-associated mutants on phospho-ERK responses were compared with the uveal melanoma-associated Q209L Gα_11_ mutation (11). Following stimulation with Ca^2+^*_o_*, the germline Gln-181 and Leu-341 mutants were revealed to have significantly (*p* < 0.001) increased maximal phospho-ERK fold-change responses (Gln-181 = 18.1 ± 1.1, Leu-341 = 18.3 ± 0.9) compared with WT Gα_11_ (14.7 ± 0.3), consistent with a gain-of-function (Fig. 4*A*). However, in the absence of Ca^2+^*_o_* stimulation, the basal phospho-ERK responses of the ADH2 mutants were demonstrated to not differ from WT Gα_11_ (Fig. 4, *A* and *B*), and thus these mutants are not constitutively activating. In contrast, the tumor-associated somatic Q209L Gα_11_ mutation led to both significantly (*p* < 0.0001) increased basal and maximal phospho-ERK fold-change responses when compared with the ADH2 mutants or WT Gα_11_, consistent with a constitutive up-regulation of MAPK signaling (Fig. 4, *A* and *B*). The effect of NPS-2143 on the phospho-ERK responses of HEK-CaSR cells expressing the ADH2-associated Gln-181 or Leu-341 mutants, or the uveal melanoma-associated Leu-209 mutant, was also assessed. NPS-2143 was added at 10 and 30 nm concentrations to cells expressing the Gln-181 and Leu-341 mutants, respectively, as these doses had rectified the Ca^2+^*_i_* responses of the Gα_11_ mutants (Fig. 2, *F* and *G*). The addition of 10 and 30 nm NPS-2143 significantly lowered the maximal fold-change responses of the Gln-181 and Leu-341 mutants to 14.0 ± 0.5 and 14.9 ± 0.4, respectively, so that these values did not differ from the phospho-ERK responses of cells expressing WT Gα_11_ (Fig. 4, *C* and *D*). However, cells expressing the uveal melanoma-associated Leu-209 mutant required NPS-2143 at a higher dose of 500 nm to successfully rectify increases in phospho-ERK responses (Fig. 4*E*).

## Discussion

Our studies demonstrate that cinacalcet and NPS-2143, which are allosteric CaSR activators and inactivators, respectively, can successfully rectify the loss-of-function associated with FHH2-causing Gα_11_ mutations and the gain-of-function associated with Gα_11_ mutations that lead to ADH2 or uveal melanomas (1, 11). Cinacalcet and NPS-2143 are allosteric modulators that are predicted to bind to the CaSR transmembrane domain (24) and influence receptor activity by altering its conformational status. These compounds have been reported to rectify the activity of FHH1- and ADH1-associated mutant CaSR proteins *in vitro* (15, 25–27). However, the ability of these agents to normalize CaSR sensitivity in the presence of an abnormality downstream of the CaSR remained unknown. The *in vitro* findings of our study indicate allosteric modulation at the level of the receptor can rectify such loss- and gain-of-function associated with mutations of the intracellular Gα_11_ protein. Indeed, these studies demonstrate that pharmacological GPCR modulation may directly overcome abnormalities affecting the downstream effector G-protein rather than by indirect effects on endogenously expressed WT G-proteins.

However, the Gα_11_ mutations showed differences in their responsiveness to allosteric CaSR modulators. For example, our study of the FHH2 mutants revealed that a 4-fold increase in the cinacalcet dose was required to normalize the loss-of-function associated with I199/200del compared withthe L135Q mutation, despite both mutations having similar EC_50_ values. Similarly, a 3-fold increase in the NPS-2143 dosage was required to rectify the gain-of-function due to the ADH2-associated F341L mutation when compared with the gain-of-function R181Q mutation, despite both mutations having similar EC_50_ values. Thus, the I199/200del and F341L mutations showed diminished sensitivity to cinacalcet and NPS-2143, respectively, and these differences in the sensitivities of the mutants to CaSR-targeted drugs may be explained by a reported crystallography study, which showed residues homologous to Ile-199 and Phe-341, in the related Gα_s_ protein to be located at the interface between GPCR and Gα-subunit (28). Thus, Gα_11_ mutations located at the GPCR-Gα interface may potentially influence the efficacy of CaSR allosteric modulators.

Cells expressing loss- and gain-of-function Gα_11_ mutants responded to nanomolar concentrations of cinacalcet (10–40 nm, which is equivalent to 3.6–14.3 ng/ml) and NPS-2143 (10–30 nm, which is equivalent to 4.4–13.3 ng/ml), respectively. However, previous *in vitro* studies of CaSR mutations leading to FHH and ADH have indicated that micromolar concentrations of these drugs may be required to rectify associated signal transduction abnormalities (25–27), and *in vivo* studies in WT rats have reported that the plasma drug concentrations of cinacalcet and NPS-2143 required to alter PTH secretion are ≥20 ng/ml and >100 ng/ml, respectively (29, 30). The responsiveness of Gα_11_ mutants to low doses of CaSR-targeted drugs may be explained by the finding that these mutants induced only minor disturbances of CaSR signal transduction. Indeed, the FHH2 and ADH2 mutants were associated with up to a 30% shift in the EC_50_ values of HEK-CaSR cells used in this study, whereas CaSR mutations leading to FHH1 and ADH1 generally cause a >50% shift in the EC_50_ value (9, 21, 31, 32). However, it remains to be established whether such low concentrations of calcimimetic and calcilytic drugs will be able to rectify *in vivo* the alterations in mineral homeostasis in FHH2 and ADH2 patients.

Somatic gain-of-function Gα_11_ mutations that induce constitutive MAPK activation have been reported in uveal melanoma and are associated with an increased likelihood of metastases (11). We therefore assessed the effects of germline ADH2-associated R181Q and F341L gain-of-function Gα_11_ mutations on MAPK signaling by measuring phospho-ERK responses. Our studies demonstrated that the ADH2 Gα_11_ mutants induced a milder increase in ERK phosphorylation when compared with the uveal melanoma Q209L Gα_11_ mutant. Moreover, up-regulation of ERK phosphorylation by the ADH2-associated Gα_11_ mutants only occurred in the presence of Ca^2+^*_o_* stimulation, and therefore these R181Q and F341L Gα_11_ mutants do not harbor constitutive activity. These findings are consistent with a recent report of an ADH2-associated R60L Gα_11_ mutation, which also enhanced MAPK activation in a non-constitutive manner (7). The finding that ADH2-associated mutations are not constitutively activating can be explained by their locations within the GTPase domain of the Gα subunit. Thus, the Gln-209 residue, which is mutated in uveal melanomas (11), is required to spatially orientate the terminal phosphate group of Gα-bound GTP (33), thereby facilitating its hydrolysis and the conversion of GTP to GDP. Mutations affecting the Gln-209 residue have been shown to abolish GTP hydrolysis, thereby leaving the Gα subunit in a permanent GTP-bound state of activation (34). In contrast, the Arg-181 and Phe-341 Gα_11_ residues_,_ which are mutated in ADH2, are not located near to the terminal phosphate of GTP, and likely induce more indirect and subtle effects on GTP hydrolysis (1). The ADH2-associated Gα_11_ mutations represent the first reports of non-constitutively activating G-protein mutations (1, 6, 7), and the milder nature of these mutations is consistent with post-natal survival, in contrast to the constitutively activating Q209L mutation, which has been shown to be cytotoxic when expressed at high levels (35), and is likely to be embryonically lethal. The occurrence of non-constitutively activating Gα_11_ mutations that are tolerated in humans and heritable, highlights the potential for such germline mutations to affect other G-proteins and be associated with disease-related phenotypes, and this possibility remains to be explored.

In summary, our studies have revealed that germline gain-of-function Gα_11_ mutations induce non-constitutive alterations in MAPK signaling, and that CaSR-targeted compounds may rectify signaling disturbances caused by germline and somatic Gα_11_ mutations, which are associated with calcium disorders and tumorigenesis, respectively. These findings indicate that allosteric modulation at the level of the receptor may influence signaling disturbances associated with mutations of the downstream G-protein.

## Author Contributions

F. M. H., M. A. N., A. C. H., and R. V. T. designed the experiments. V. N. B., S. A. H., and N. R. performed the Ca^2+^*_i_* measurement experiments. C. M. G. performed the siRNA knockdown experiments. V. N. B. performed the ERK phosphorylation experiments. J. H. and A. M. S. prepared and supplied the NPS-2143 compound. F. M. H., V. N. B., C. M. G., and R. V. T. wrote the manuscript. All authors reviewed the results and approved the final version of the manuscript.

## Footnotes

4 The abbreviations used are:

G-protein: guanine nucleotide-binding protein
ADH: autosomal dominant hypocalcemia
AUC: area under the curve
Ca^2+^*_i_*: intracellular calcium
[Ca^2+^]*_i_*: intracellular calcium concentration
Ca^2+^*_o_*: extracellular calcium
[Ca^2+^]*_o_*: extracellular calcium concentration
CaSR: calcium-sensing receptor
EC_50_: half-maximal effective concentration
FHH: familial hypocalciuric hypercalcemia
FRT: FLP recombination target
Gα_11_: G-protein α-11
Gαs: G-protein α-s
GPCR: G-protein-coupled-receptor
HBSS: Hank's balanced salt solution
Indo-1-AM: indo-1-acetoxymethylester
m: mutant
PTH: parathyroid hormone.
